# The role of mitophagy during oocyte aging in human, mouse, and Drosophila: implications for oocyte quality and mitochondrial disease

**DOI:** 10.1530/RAF-21-0060

**Published:** 2021-10-11

**Authors:** Rachel T Cox, Joanna Poulton, Suzannah A Williams

**Affiliations:** 1 Department of Biochemistry and Molecular Biology, Uniformed Services University, Bethesda, Maryland, USA; 2 Nuffield Department of Women’s & Reproductive Health, University of Oxford, Oxford, UK

**Keywords:** mitophagy, mitochondria, ovary, oocyte, Drosophila, mouse, human, mtDNA, ARTs

## Abstract

There is a worldwide trend for women to have their first pregnancy later in life. However, as oocyte quality declines with maternal aging, this trend leads to an increase in subfertility. The cellular mechanisms underlying this decline in oocyte competence are poorly understood. Oocyte mitochondria are the subcellular organelles that supply the energy that drives early embryogenesis, and thus their quality is critical for successful conception. Mitochondria contain their own DNA (mtDNA) and mutations in mtDNA cause mitochondrial diseases with severe symptoms, such as neurodegeneration and heart disease. Since mitochondrial function declines in tissues as humans age accompanied by an accumulation of mtDNA mutations, mtDNA is implicated as a cause of declining oocyte quality in older mothers. While this mutation load could be caused by declining accuracy of the mitochondrial replisome, age-related decline in mitochondrial quality control likely contributes, however knowledge is lacking. Mitophagy, a cellular process which specifically targets and recycles damaged mitochondria may be involved, but studies are scarce. And although assisted reproductive technologies can help older mothers, how these techniques affect the mechanisms that regulate mitochondrial and oocyte quality have not been studied. With the long-term goal of understanding the molecular mechanisms that control mitochondrial quality in the oocyte, model systems including Drosophila and mouse as well as human oocytes have been used. In this review, we explore the contribution of mitophagy to oocyte quality and the need for further systematic investigation in oocytes during maternal aging using different systems.

**Lay summary:**

Mitochondria are small parts of cells called organelles that generate the chemical energy needed for life. Hundreds of thousands of mitochondria in the developing eggs of the mother support the initial growth and development of the fertilized egg. However, due to increasingly diminished function over time, mitochondria generate less energy as we age, posing real problems for older women considering pregnancy. It is possible that this declining energy could be responsible for declining fertility as women age. Energy may decline because mitochondria fail and the cell’s way of keeping them healthy become less efficient as we age. This review summarizes what is known about mitochondrial quality control in developing eggs as they age. In the future, understanding how the best mitochondria are selected and maintained in the egg, and hence the future baby, may enable older women with or without mitochondrial problems, to have healthy children.

## Introduction

Our understanding of human reproduction and fertility has greatly increased in the last two decades. This is due to the knowledge gleaned from experimental model systems and through advances in assisted reproductive technologies (ART). Female fertility rates decline with age. Concurrently, women in developed countries are increasingly having children later in life (O’Brien & Wingfield 2019). However, many of the underlying intrinsic cellular conditions that potentially influence whether each fertilized egg will result in a healthy embryo are still not understood.

**Figure 1:**
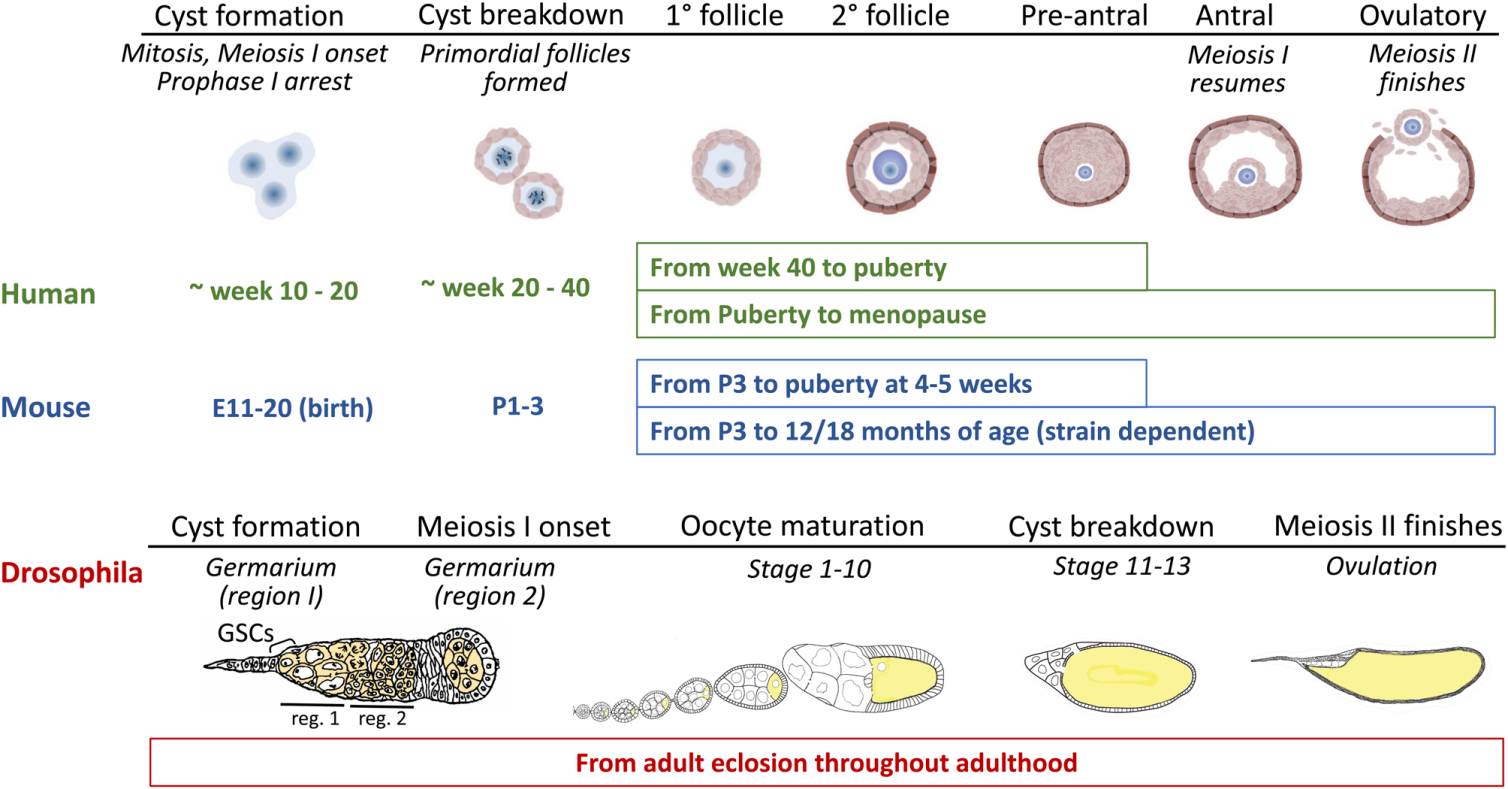
Timing and conserved aspects of oocyte development in humans, mouse, and Drosophila. Human, mouse, and Drosophila undergo female germ cell cyst formation, cyst breakdown, and oocyte maturation and development. Human and mouse germ cell cyst and follicle stages are indicated along the top. Birth occurs at ~week 40 of fetal development in humans (green) and embryonic (E) day 20 for mouse (blue). Drosophila (red) shares many cell biological similarities with human and mouse such as cyst breakdown and oocyte maturation. In Drosophila, germline stem cell division, cyst formation, and oocyte development occur throughout adulthood.

The mature egg is the largest cell in the body, and the cytoplasm contains everything required for early embryo development apart from the paternal DNA contributed by the sperm. Thus, at fertilization, all the energetic and nutritional requirements for development of the embryo prior to implantation are derived from the egg. Mature ovulated eggs are generated from primordial germ cells (PGCs) by processes named 'oocyte development' in mammals and 'oogenesis' in Drosophila (Fig. 1). The process is similar but not identical in these very different organisms: the oocytes develop from what is believed to be a finite pool in the majority of mammals, whereas in Drosophila, the oocytes arise from germline stem cells (GSCs) (Matova & Cooley 2001). The process of oocyte development is a complex and dynamic process, requiring intricate interactions between different cells within the gonad as well as appropriate endocrine support. The oocyte increases in size from just a few microns in diameter to around 100 mm in humans, around 70 µm in mouse, and 0.5 mm in Drosophila. The oocyte was believed to be a bystander in this process for many years. However, ablation of oocyte-specific growth and differentiation factor 9 (GDF9) in mouse in 1996 revealed the oocyte as the master conductor in driving the development of the follicle and hence its own destiny (Dong *et al.* 1996). Likewise, the Drosophila oocyte is responsible for signaling to the surrounding somatic follicle cells to establish the body pattern of the resulting embryo (Lehmann 1995).

Studies on human ovarian tissue are clearly required to understand human ovarian function. However, experiments interrogating human ovarian function are inherently limited by tissue quantity, type (e.g. age), and source (e.g. patients in poor health or cadavers). In addition, experiments need to be carried out *ex vivo*. To enable us to explore ovarian function and development, model species have considerable advantages for elucidating the cellular processes underlying oocyte quality. Mice have been a model laboratory species for many years and undergone extensive investigation as they are small, versatile, and reproduce quickly, that is, fertilization to breeding in 9 weeks. Many transgenic mouse models have been created, made possible by advances in genetic modification, enabling targeted research into ovarian function as well as oocyte and embryo development (Barnett *et al.* 2006). Drosophila has been used as a model for over 100 years. Although Drosophila clearly differs in body plan compared to mice, deciphering the intricacies of cellular signaling pathways and molecular interactions has been advanced using Drosophila in ways not achievable with mouse models due to the speed of reproduction (7 days from germline stem cell to mature egg), the array of genetic tools available, and the accessibility of the ovaries.

Mitochondria are small subcellular organelles that provide cells with the chemical energy ATP. Growth and development of the oocyte demands highly functional mitochondria to support not only its own development, but also to ensure the final product, the mature egg, is commensurate to support early embryonic development. After fertilization, the zygote, as it is now known, embarks on a number of developmental stages resulting from multiple subsequent cell divisions. Critically, all energetics driven by the mitochondria are derived from the oocyte. Thus, in mammals and Drosophila, the generation and maintenance of high functioning mitochondria by the oocyte during development is crucial for early embryo development.

Mitochondrial numbers and dynamics change during oocyte development in mammals and Drosophila (see below). While there is some indication of decreased mitochondrial quality and function with age (Diot *et al.* 2016*b*), there is still much that we do not understand. There is a wide-spread appreciation of the requirement for ‘good’ mitochondria in oocytes, especially in the case of inherited mitochondrial disease (Poulton *et al.* 2010), and there are still many questions unanswered about the mechanisms that regulate this process within the oocyte and how this occurs during oocyte development and aging. One potential mechanism regulating mitochondrial quality during oocyte development, which may be affected by age, is mitophagy. Mitophagy is the process by which damaged mitochondria are targeted for destruction (Box 1). There are currently several known pathways regulating this process, however, new pathways are certain to emerge. This review summarizes the current knowledge of germ cell mitophagy using humans and two model species: mouse and Drosophila. The role of mitophagy during oocyte development and aging has not been extensively explored and yet is critical to understand. Knowledge derived from each model improves our ability to investigate mitochondrial function in human oocyte development.

**Box 1** Summary of mitophagy pathways

**Figure 2:**
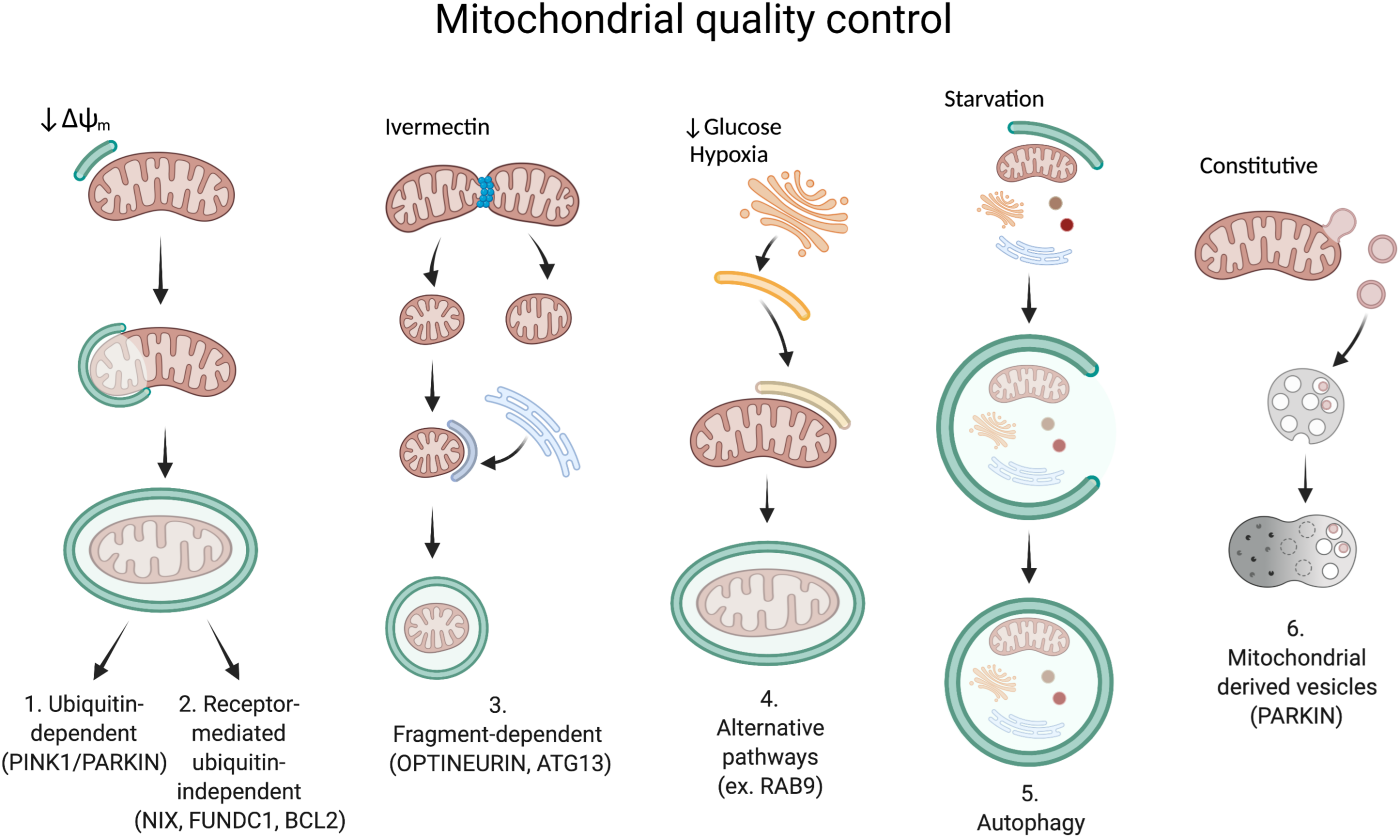
Mitochondrial quality control pathways. Mechanisms governing mitochondrial turnover can be subdivided into six major pathways (see Box 1 for further detail). Representative cellular cues that stimulate each pathway are at the top and examples of the process and the proteins required for each pathway are listed at the bottom. Pathways 1–5 ultimately result in the mitochondrion engulfed in an autophagosome membrane (green) that will fuse with the lysosome, destroying the organelle. The source of this membrane differs depending on the pathway (pathways 3 and 4) and in some instances has not yet been identified (pathways 1 and 2). Mitochondria are also turned over by non-selective autophagy (pathway 5). Mitochondrial derived vesicles turn over small portions of the mitochondrion (Sugiura *et al.* 2014) by transporting them to the lysosome, arguably distinct from mitophagy because autophagosomes are not involved (pathway 6). RAB9-dependent alternative/non-canonical mitophagy involves autophagosomes but not ATG5 or 7 (Arakawa *et al.* 2017). Figure created with BioRender.

Mitophagy shares much of the core machinery used by general autophagy. Mitophagy can be triggered by several different stimuli and there are likely pathways that have yet to be identified. Mitochondrial fragmentation often takes place before the organelle is degraded; thus, proteins controlling mitochondrial fission appear integral to the process and often interact with specific mitophagy components (Xian & Liou 2021). The descriptions are shown below (Fig. 2). The mitophagy pathway described originally involves PINK1 and PARKIN proteins (pathway 1) (reviewed in Pickles *et al.* 2018). Once a mitochondrion loses its mitochondrial membrane potential (Δψ_m_), PINK1 (PTEN-induced kinase 1) is stabilized at the outer membrane, then phosphorylates the ubiquitins on various proteins. This signal recruits the cytoplasmic protein PARKIN (an E3 Ubiquitin ligase) to ubiquitinylate additional sites on proteins on the outer membrane. Subsequently, receptors are then recruited to the organelle and serve as a cue for degradation. The lipidated adaptor protein LC3 located on isolation membranes (also called phagophores) (green) binds to the receptors. This membrane grows and envelopes the damaged organelle, ultimately fusing with the lysosome for degradation. A second related pathway involves FUNDC1/NIX/BCL2 (pathway 2) (reviewed in Chen *et al.* 2020*a*). This pathway is triggered by loss of Δψ_m_, as well as hypoxia (NIX/BLC2). NIX has been best characterized for mitochondrial clearance during red blood cell maturation (Sandoval *et al.* 2008). One difference is that FUNDC1, NIX, and BCL2 are outer membrane receptors that directly bind to LC3 and recruit an isolation membrane, thereby bypassing any need for ubiquitinylation (reviewed in Zachari & Ktistakis 2020). An additional pathway was recently identified that is ubiquitin-dependent but acts independently of PINK1/Parkin (pathway 3) (reviewed in Zachari & Ktistakis 2020). Exposing cells to the lactone ivermectin causes mitochondrial damage and fragmentation (Zachari *et al.* 2019). Once this happens, the E3 Ubiquitin ligases TIAF2, CIAP1, and CIAP2, conjugate ubiquitin on the mitochondrial membrane and ultimately recruit receptors (Optineurin, Atg13). The isolation membrane that subsequently binds to these receptors appears to originate from the endoplasmic reticulum (ER) (blue), unlike for pathways 1 and 2 (Zachari *et al.* 2019). An alternative non-canonical pathway that has been identified involves RAB9 positive vesicles or membranes (pathway 4) (reviewed in Zachari & Ktistakis 2020). This pathway does not appear to require LC3 lipidation, nor is it known whether ubiquitinylation or receptors are involved (reviewed in Zachari & Ktistakis 2020). In this pathway, phosphorylation of RAB9 stimulates mitochondrial fission (Saito *et al.* 2019). Subsequently, isolation membranes derived from the trans-Golgi network (yellow) engulf the damaged mitochondrion (Saito *et al.* 2019). In addition to specific mitophagy pathways, bulk autophagy also degrades mitochondria (pathway 5) (reviewed in Lahiri *et al.* 2019). Bulk autophagy and its effect on cell physiology and disease is a well-studied area. In response to stimuli such as starvation, an isolation membrane forms (green) and surrounds a part of the cytoplasm, thus scooping up multiple cellular components. Once membrane formation is complete, the autophagosome fuses with the lysosome in order to degrade and recycle the components for cellular use (Lahiri *et al.* 2019). A final pathway that does not involve whole organelle destruction involves mitochondrial-derived vesicles (MDVs) (pathway 6) (reviewed in Sugiura *et al.* 2014). This pathway appears to happen constitutively in the cell and may be important for normal mitochondrial homeostasis. Small MDVs containing oxidized mitochondrial proteins bud off individual mitochondria then fuse with endolysosomes for degradation in the lysosome (Soubannier *et al.* 2012*a*,*b*). This mechanism relies on PINK1 and PARKIN (McLelland *et al.* 2014).

## Species comparison of mitochondrial changes during oocyte development

In human, mouse, and Drosophila, female germ cells undergo incomplete mitoses to form interconnected cysts which have been shown to have the capacity to share cellular contents, including mitochondria (de Cuevas *et al*. 1997, Pepling & Spradling 1998, Pepling *et al.* 1999). In human and mouse, cyst formation takes place during fetal development, then subsequently break down to form a pool of single primordial follicles with many undergoing apoptosis around birth. In Drosophila, cyst formation and breakdown occur continuously in adults, thus all stages can be viewed simultaneously. Mouse and human oocyte development have comparable developmental stages (Fig. 1). Mice reach puberty at around 4–5 weeks of age, whereas in humans, puberty occurs at approximately 12 years of age. After puberty, preantral follicles have the endocrine support required to enable growth and maturation into an ovulatory follicle; although many undergo apoptosis during the process of follicle development. As the follicle completes development, the oocyte resumes meiosis and undergoes two successive divisions that give rise to polar bodies. Follicle development and ovulation occur throughout a woman’s reproductive years terminating at menopause which occurs around 50 years of age on average.

### Germ cell mitochondria in mammals and Drosophila

Mitochondrial shape, numbers, activity, localization, and ultrastructure have been examined by transmission electron microscopy (TEM) and fluorescent microscopy in human, mouse, and Drosophila germ cells and oocytes. In Drosophila, mitochondria were originally described during oogenesis using TEM (reviewed in Mulligan 2003). Immunofluorescence offered a more comprehensive examination of numbers, shape, and location.

During cyst formation in the *Drosophila germarium* (Fig. 1), mitochondria remain fragmented as the germ cells undergo rapid mitoses to form the 16-cell cyst (Cox & Spradling 2003). Once cyst formation is complete, the mitochondria associate with a germline-specific ER-derived organelle called the fusome. As the oocyte is specified from one of the 16 germ cells in the cyst, a subset of mitochondria from the 15 connected nurse cells moves into the oocyte using microtubules and their associated motors to form the Balbiani body, an organelle-rich body found in most oocytes (Cox & Spradling 2006). As the oocyte continues to mature, the connected sister germ cells stop transferring mitochondria, in while the oocyte’s own mitochondria undergo biogenesis to replicate. Late in oogenesis, the nurse transport their cytoplasmic contents, including mitochondria, into the oocyte and subsequently undergo apoptosis (de Cuevas *et al.* 1997).

In mouse early embryos, TEM and northern dot blot analysis of mitochondrial rRNA and mRNA estimated that there is approximately one copy of mitochondrial DNA (mtDNA) per mitochondrion (Piko & Matsumoto 1976) (Piko & Taylor 1987). Increased sophistication of imaging techniques makes it possible to use fluorescence microscopy to get a full picture of entire oocytes. However, given the large size of the growing oocyte (~80 µm mice, ~100 µm human) and the heterogeneous mitochondrial distribution, acquiring accurate mitochondrial numbers can be challenging. In mouse and humans, the number of mitochondria reaches a plateau during oocyte development and the organelles do not resume biogenesis until after embryo implantation. Low level mtDNA turnover has however been documented in mice (McConnell & Petrie 2004), and increased copy number and transcription in cows (May-Panloup *et al.* 2005). There are approximately 100–200 mitochondria in PGCs in mouse and human. These mitochondria are spherical and have reduced cristae. During oocyte development, this number increases and reaches over 100,000 in the mature oocyte (Cao *et al.* 2007, Wai *et al.* 2008). In addition to a large increase in numbers, there are also general changes to mitochondrial localization and activity. In mouse and human PGCs and primary follicles, mitochondria associate with the ER and the Balbiani body (Motta *et al.* 2000, Pepling *et al.* 2007). In the developing oocyte, mitochondria are mostly homogeneously localized throughout the cytoplasm, however, the mitochondria located at the plasma membrane have higher membrane potential and thus may be more active (Van Blerkom 2009). In oocytes from women and mice of advanced maternal age, this membrane potential is greatly decreased and thus indicative of lower activity (Pasquariello *et al.* 2019).

### The effect of oxidative stress on oocytes

As mitochondria provide important metabolites and energy, and contain their own error-prone DNA, understanding the cell biological mechanisms governing mitochondrial location and function in germ cells has been recognized as critical to understanding oocyte fitness and almost certainly affects oocyte quality during maternal aging. Pyruvate oxidation is essential for oocyte development in mouse (Johnson *et al.* 2007). In contrast, ovulated oocyte and preimplantation embryo metabolism is based on low levels of oxidation of pyruvate, lactate, and specific amino acids, followed by a dramatic increase in oxidative phosphorylation at blastocyst formation (Gardner & Wale 2013). Likewise, in Drosophila, during the majority of oogenesis, germ cells rely on oxidative phosphorylation (Sieber *et al.* 2016). The first half of embryogenesis is also heavily reliant on oxidative phosphorylation, after which a switch to glycolysis occurs in preparation for the exponential growth experienced during larval growth (Tennessen *et al.* 2011).

Oocytes in mouse and human primarily use oxidative phosphorylation for their energy demands, with oxygen consumption low in the zygote and first few cell divisions and increasing several folds at the blastocyst stage (Leese 2012). This burst requires a robustly functioning electron transport chain (Dumollard *et al.* 2007*a*,*b*, Dalton *et al.* 2014). However, electron transport is the main source of mitochondrial oxidative damage due to the inevitable production of reactive oxygen species (ROS). Oxidative stress is associated with maternal aging and supplementing aging mice with the antioxidant CoenzymeQ can preserve ovarian reserve (Ben-Meir *et al.* 2015). Since the resumption of meiosis is an energy-intensive process, it is perhaps not surprising that impaired mitochondrial function can lead to chromosomal aberrations. Aneuploidy greatly increases with age with up to 60% of oocytes exhibiting aneuploidy after age 40 (Angell 1994, Pellestor *et al.* 2003). Oocytes retrieved from women with advanced maternal age have decreased mitochondrial function associated with increased oxidative stress and spindle abnormalities (Pasquariello *et al.* 2019). In addition, there is evidence that treating mouse and human oocytes with compounds that ameliorate ROS and their subsequent damage can improve chromosome segregation (Pasquariello *et al.* 2019, Al-Zubaidi *et al.* 2021). Reduced numbers of mitochondria (Reynier *et al.* 2001) and mtDNA (Wai *et al.* 2010) may also play a role.

In Drosophila, females mutant for *Superoxide Dismutase 2* (*SOD2*), the ROS scavenger in mitochondria, only live for <24 h, have greatly reduced ATP levels and increased oxidative damage (Sen *et al.* 2013). While this is insufficient time for egg laying to start, mitochondria in the developing follicles are abnormally localized and clumped, indicative of stress (Sheard *et al.* 2020). Together, these observations give strong support that maintaining undamaged and fully competent mitochondria during oocyte development is critical for healthy oocytes.

### Germ cell mitochondrial changes during aging

The mechanisms underlying ageing have been discussed for decades. Advances in molecular and cell biology of the last three decades have identified multiple potential mechanisms that contribute to aging. It has become clear that mitochondrial function, which plays a major role in many cellular pathways including ATP production, nuclear gene expression and epigenetic alterations, declines with age. In Drosophila, mitochondrial fission increases with age in GSCs, and if fission is genetically increased, GSCs are lost more frequently from the stem cell niche (Amartuvshin *et al.* 2020). Conversely, they found increasing fusion makes GSCs more competitive for remaining in the niche. How mitochondrial dynamics changes with age has not been examined in more developed follicles in Drosophila.

In humans, declining oocyte potential is strongly associated with oocyte karyotypic instability in older women. Both chaotic mosaicism and morphologically abnormal meiotic apparatus are significantly associated with low mitochondrial membrane potential in preimplantation embryos (Wilding *et al.* 2003). Oocytes retrieved from women of increased maternal age who were being treated for idiopathic infertility showed structural and functional mitochondrial deficits (Muller-Hocker *et al.* 1996, Chan *et al.* 2005, Murakoshi *et al.* 2013). Mitophagy is one of the mechanisms for maintaining mitochondrial quality that appears to decline in aging. While the quality and the quantity of mtDNA are directly implicated in oocyte function, their importance in aging and the role of mitophagy remain unclear.

## Mitophagy as a potential driver of oocyte quality

### Mitophagy mechanisms

Superfluous or damaged mitochondria are recycled within the cell by either bulk-autophagy, or the more selective mitochondria specific process, mitophagy (Fig. 2 and Box 1, reviewed in Pickles *et al.* 2018, Zachari & Ktistakis 2020, Doblado *et al.* 2021). However, the developmental *in vivo* contexts for mitophagy are poorly understood. While the molecular details of some pathways, such as the best characterized PINK1/Parkin pathway (Fig. 2, pathway 1), are well understood (Narendra *et al.* 2010), others are still poorly defined. PINK1 and PARK2 (Parkin) are mutated in inherited forms of early onset Parkinson’s disease. This pathway causes ubiquitination of mitochondrial proteins, triggering formation of a specialized membrane called a phagophore (Fig. 2, green), which engulfs the mitochondrion forming an autophagosome (Narendra *et al.* 2010). In contrast, pathways mediated by receptors on the mitochondrial outer membrane, such as NIX, which functions specifically during red blood cell development (Fig. 2, pathway 2) trigger mitophagy independently of ubiquitination (Sandoval *et al.* 2008). These receptors bind to the adaptor protein LC3 that characterizes autophagosomes that then engulf their target. While the receptors and source of the phagophore membrane differ, the final downstream steps to degrade the organelles are often shared (Fig. 2). Two other pathways have been identified that are PINK1/PARK independent (Fig. 2, pathways 2 and 4). Once the autophagosome forms (pathways 1–5), it fuses with a lysosome and its contents are degraded at low pH. For cell biological studies, a key mitophagy readout is co-localization of mitochondria with autophagosomes or lysosomes. Finally, a unique quality control pathway that uses mitochondrial derived vesicles can recycle small portions of the mitochondrion for direct degradation by the lysosome (Fig. 2, pathway 6) (Sugiura *et al.* 2014). Please see other literature for more detail (Pickles *et al.* 2018, Zachari & Ktistakis 2020, Doblado *et al.* 2021) and for RAB9-dependent alternative/non-canonical mitophagy (Arakawa *et al.* 2017) and Table 1 (adapted from other publications (Dickinson *et al.* 2016, Zachari & Ktistakis 2020)) for essential genes.

**Table 1:** Known female germ cell phenotypes for autophagy/mitophagy genes.

Process/protein^a^		Species	Mouse fertility^b^	Fly fertility	Mouse viability^b^
Turning on the pathway					
mTORC1	Many	H/M/D	Reduced fertility^1^	Tor RNAi-sterile	
TFEB	Transcription factor	H/M/D	ND	ND	Lethal^2^
RHEB	Small GTP-binding proteins, Ras superfamily	H/M/D	Fertile^3^	Female sterile (viable mutation)^4^	Embryonic lethal^5^
Initiation of autophagosome biogenesis					
ULK complex					
ULK1, 2	Ser/Thr kinase catalytic subunit of ULK	H/M/D	ND	(Atg1) Female sterile (RNAi)^6, 7^	DKO for ULK1/2 Embryonic lethal^8^
ATG13	Regulatory subunit of ULK	H/M/D	ND	Fertile	lethal^9^
ATG101	Subunit of ULK	H/M/D	ND	ND	ND
FIP200	Subunit of ULK	H/M/D	ND	(Atg17) ND	Embryonic lethal^10^
AMPK (α, β, γ)	Ser/Thr kinase	H/M/D	(α1) Decreased litter size, abnormal mitochondrial physiology^11^	Female sterile (RNAi)^12^	Viable (α1, 2)^13^ Viable (γ)^13^
ATG9	Transmembrane protein	H/M/D	ND	Sterile (null mutant)^14^	Lethal neonatal^15^
VPS34-I					
VPS34	Class III PI-3 kinase subunit of VPS34-1	H/M/D	ND	ND	Embryonic lethal^16^
VPS15	Subunit of VPS34-1	H/M/D	ND	Semi-sterile (RNAi)^12^	Embryonic lethal^17^
BECN1	Regulatory subunit of VPS34-1	H/M/D	Infertile^18^	(Atg6) ND	Embryonic lethal^19^
ATG14L	Subunit of VPS34-1	H/M/D	ND	ND	ND
AMBRA	Regulator of BECN1	H/M	ND	–	Lethal^20^
MAPKAP2,3	Ser/Thr kinases	H/M/D	Fertile^21^	Semi-sterile (RNAi)^12^	Viable^21^
DFCP1	PI(3)P-binding protein	H/M	ND	–	Viable^22^
WIPI1,2	PI(3)P-binding protein	H/M/D	ND	ND	ND
Building the autophagosome					
Lipidation complex					
ATG12	Part of ubiquitin ligation-like (E3) complex	H/M/D	ND	ND	Lethal neonatal^23^
ATG3	Part of ubiquitin ligation-like (E3) complex	H/M/D	ND	ND	Lethal neonatal^24^
ATG5	Part of ubiquitin ligation-like (E3) complex	H/M/D	Oocyte development normal, early embryonic lethal^25^	Fertile	Lethal neonatal^26^
ATG7	Part of ubiquitin ligation-like (E3) complex	H/M/D	Subfertile^27^	ND	Lethal neonatal^28^
ATG16L	Part of ubiquitin ligation-like (E3) complex	H/M/D	ND	Fertile	Lethal neonatal^29^
LC3A, B, C	Ubiquitin-like proteins	H/M/D	LC3B Fertile (JAX)	(Atg8a, Atg8b) ND	LC3B viable^30^
GABARAP, L1, L2	Ubiquitin-like proteins	H/M/D	ND	(Atg8a, Atg8b) ND	GABARAP viable^31^ L1 viable^32^ L2 lethal^33^
ATG2b	Phospholipid binding/transfer protein	H/M/D	ND	ND	Viable (IMPC)
VMP1	ER-resident protein	H/M/D	ND	ND	Lethal^34^
Fusing autophagosome with lysosome					
STX17	SNARE protein	H/M/D	ND	(Syx17) fertile	ND
RAB7	Small GTP-binding proteins, Ras superfamily	H/M/D	ND	ND	s
EPG5	RAB7 effector protein	H/M/D	ND	ND	Viable, reduced survival ^36^
HOPS (VPS11, VPS16, VPS18, VPS33A)	Tethering complex	H/M/D	ND	(Car) ND (Cm) ND	VPS33A, 16, viable, impaired motor function^37^
PLEKHM1	HOPS-interacting protein	H/M/D	Fertile ^38^	ND	Viable^38^
Ubiquitin-mediated mitophagy					
PINK1	Kinase	H/M/D	Fertile (JAX)	Sterile^39^	Viable^40^
OPTN	Mitophagy receptor	H/M/D	Fertile^41^	(Nemo) Semi-sterile ^7^	Viable^41^
PARK2	E3 ubiquitin ligase	H/M/D	Fertile^42^	Semi-sterile ^12, 43^	Viable^42^
P62 (SQSTM1)	Mitophagy receptor	H/M/D	ND	(Ref(2)P) Fertile	Viable^44^
TAX1BP1	Mitophagy receptor	H/M	ND	–	Viable^45^
NDP52	Mitophagy receptor	H/M	ND	–	Viable (IMPC)
NBR1	Mitophagy receptor	H/M	ND	–	Viable (IMPC)
OMM mitophagy receptors				–	
BNIP3	Mitophagy receptor	H/M	Fertile^46^	–	Viable^46^
BNIP3L (NIX)	Mitophagy receptor	H/M	ND	–	Viable^47^
BCL2L13	Mitophagy receptor	H/M	Reduced fertility ^48^	–	Viable^48^
FUNDC1	Mitophagy receptor	H/M	Fertile^49^	–	Male lethal (IMPC)
Lysosomal					
LAMP1		H/M/D	Fertile^50^	ND	Viable^50^

^a^Protein functions listed are those that are related to mitophagy/autophagy. Other important cellular functions may have been ascribed to individual proteins. ^b^Fertility and viability were assessed from literature, Jackson Laboratory (JAX) breeding information, Mouse Genome Informatics (MGI), and the International Mouse Phenotyping Consortium (IMPC). Fertility information may indicate that homozygotes can breed or produce offspring however this does not necessarily mean oocyte development in normal. In some cases, while a strain is viable it may have abnormalities and it is not clear if it is fertile (ND). Viability was assessed for available information on whole body knockout. Phenotypic description of whole body knockouts may not be included in the original study generating the knockout strain. DKO, double knockout; H/M/D, human/mouse/Drosophila; ND, no data; OMM, outer mitochondrial membrane. ^1^Guo and Yu (2019); ^2^Steingrimsson *et al.* (1998); ^3^Baker *et al.* (2014); ^4^Stocker *et al.* (2003); ^5^Goorden *et al.* (2011); ^6^Lieber *et al.* (2019); ^7^Kuhn *et al.* (2015); ^8^Cheong *et al.* (2014); ^9^Kaizuka and Mizushima (2016); ^10^Gan *et al.* (2006); ^11^Bertoldo *et al.* (2015); ^12^Sopko *et al.* (2014); ^13^Jorgensen *et al.* (2004); ^14^Wen *et al.* (2017); ^15^Saitoh *et al.* (2009); ^16^Zhou *et al.* (2011); ^17^Nemazanyy *et al.* (2013); ^18^Gawriluk *et al.* (2011); ^19^Yue *et al.* (2003); ^20^Fimia *et al.* (2007); ^21^Ronkina *et al.* (2007); ^22^Zhong *et al.* (2020); ^23^Malhotra *et al.* (2015); ^24^Sou *et al.* (2008); ^25^Tsukamoto *et al.* (2008*b*); ^26^Kuma *et al.* (2004); ^27^Song *et al.* (2015); ^28^Komatsu *et al.* (2005); ^29^Saitoh *et al.* (2008); ^30^Cann *et al.* (2008); ^31^O’Sullivan *et al.* (2005); ^32^Sasai *et al.* (2017); ^33^Skarnes *et al.* (2011); ^34^Morishita *et al.* (2019); ^35^Kawamura *et al.* (2012); ^36^Zhao *et al.* (2013); ^37^Zhen and Li (2015); ^38^Fujiwara *et al.* (2016); ^39^Politi *et al.* (2014); ^40^Kitada *et al.* (2007); ^41^Slowicka *et al.* (2016); ^42^Itier *et al.* (2003); ^43^Cox and Spradling (2009); ^44^Wada *et al.* (2006); ^45^Iha *et al.* (2008); ^46^Diwan *et al.* (2007); ^47^Yuan *et al.* (2017); ^48^D’Alonzo and Hong (2017); ^49^Zhang *et al.* (2016); ^50^Andrejewski *et al.* (1999).

### The effect of mitophagy gene loss on oocyte development and fertility

Mitophagy has the potential to remove mtDNA mutants during oogenesis and oocyte development due to its specificity removing damaged mitochondria. While there are several pathways known to regulate mitophagy, which ones play a role during oogenesis and oocyte development has not been fully investigated.

The Drosophila genome has excellent homology to the human genome, with over 75% of known human diseases having a homolog in flies (Bier 2005). Thus, mutants or RNAi knockdown have been analyzed for many of the known genes involved in mitophagy and autophagy (Table 1). If mutations in a gene are non-viable, it is straight-forward to use germ cell specific RNAi to identify fertility defects. Of those genes that have been examined, many cause female sterility or semi-sterility when lost in the germline (Table 1). However, most of the genes involved in autophagy and mitophagy have not been examined for their effect on mitochondrial quality and turnover in Drosophila female germ cells. The PINK1/Parkin pathway is required for fertility in Drosophila and mutants in either gene have clumped, mislocalized germ cell mitochondria that are hyperfused. However, Parkin and PINK1 do not appear to alter mtDNA inheritance (see below).

Autophagy is essential for normal preimplantation development in mice and components of the autophagy pathway have been knocked out in mouse models. In general, the resulting phenotypes indicate mice lacking the proteins required for the pre-conjugation system die very early in embryogenesis and whereas the mice lacking the proteins required for conjugation die either at birth or surviving to adulthood (reviewed in Kuma *et al.* 2017). A germline-specific knockout of *Atg5* is fertile but the resulting embryos do not develop past the embryonic four to eight cell stages (Tsukamoto *et al.* 2008*b*). Using LC3 to identify autophagosomes, Meng *et al.* showed that autophagy is a key process in preantral follicular atresia, while antral follicles degenerate mostly through apoptosis (Meng *et al.* 2018).

Global knockout of several key autophagy and mitochondrial dynamics genes, including *Beclin1*, *Atg7*, *Opa1*, *Mfn1*, and *Mfn2* are embryonic lethal. BECLIN1 is an essential autophagy protein involved in autophagosome formation. Mice that are homozygous for *Beclin1* knockout die as embryos (Qu *et al.* 2007) and heterozygotes have a shortened lifespan and increased risk of mouse mammary cancer (Vega-Rubin-de-Celis 2019). When targeted to mouse ovary, deficiency of *Beclin1* results in 56% fewer primordial follicles at postnatal day 1 (Gawriluk 2014). Constitutively, active *Beclin1* demonstrates the central importance of mitophagy, because by increasing autophagy it can rescue a model of mouse aging that has impaired fertility and mitochondrial function (Murata *et al.* 2009, Fernandez *et al.* 2018). Oocyte-specific deletion of *Mfn1*, but not *Mfn2*, results in a complete loss of oocyte growth and ovulation due to a block in folliculogenesis at the preantral-to-antral follicle transition (Carvalho *et al.* 2020). In addition, *Mfn1* knockdown mice exhibit abnormal mitochondrial clustering and enlarged mitochondria with disrupted cristae. Together, this work demonstrates that the balanced expression of modulators of mitochondrial dynamics is critical for proper oocyte development.

With aging, *Pink1* mutant mice have increased mitochondrial neuronal deficits (Gispert *et al.* 2009). However, it is well established that mice mutant for *Pink1* and *Parkin* do not normally display characteristic Parkinson’s disease neuronal loss, nor are they infertile (Goldberg *et al.* 2003, Perez & Palmiter 2005, Kitada *et al.* 2007). Recent literature has demonstrated Parkinson’s disease phenotypes, including loss of dopaminergic neurons, after stress induced by exhaustive exercise or intestinal infection (Sliter *et al.* 2018, Matheoud *et al.* 2019). Neither fertility nor oocyte development were examined under these conditions and thus need to be explored.

## Control of mtDNA mutation load during oogenesis and oocyte aging: implications for mitochondrial disease

### Importance of the developmental genetic bottleneck in mtDNA transmission

**Figure 3:**
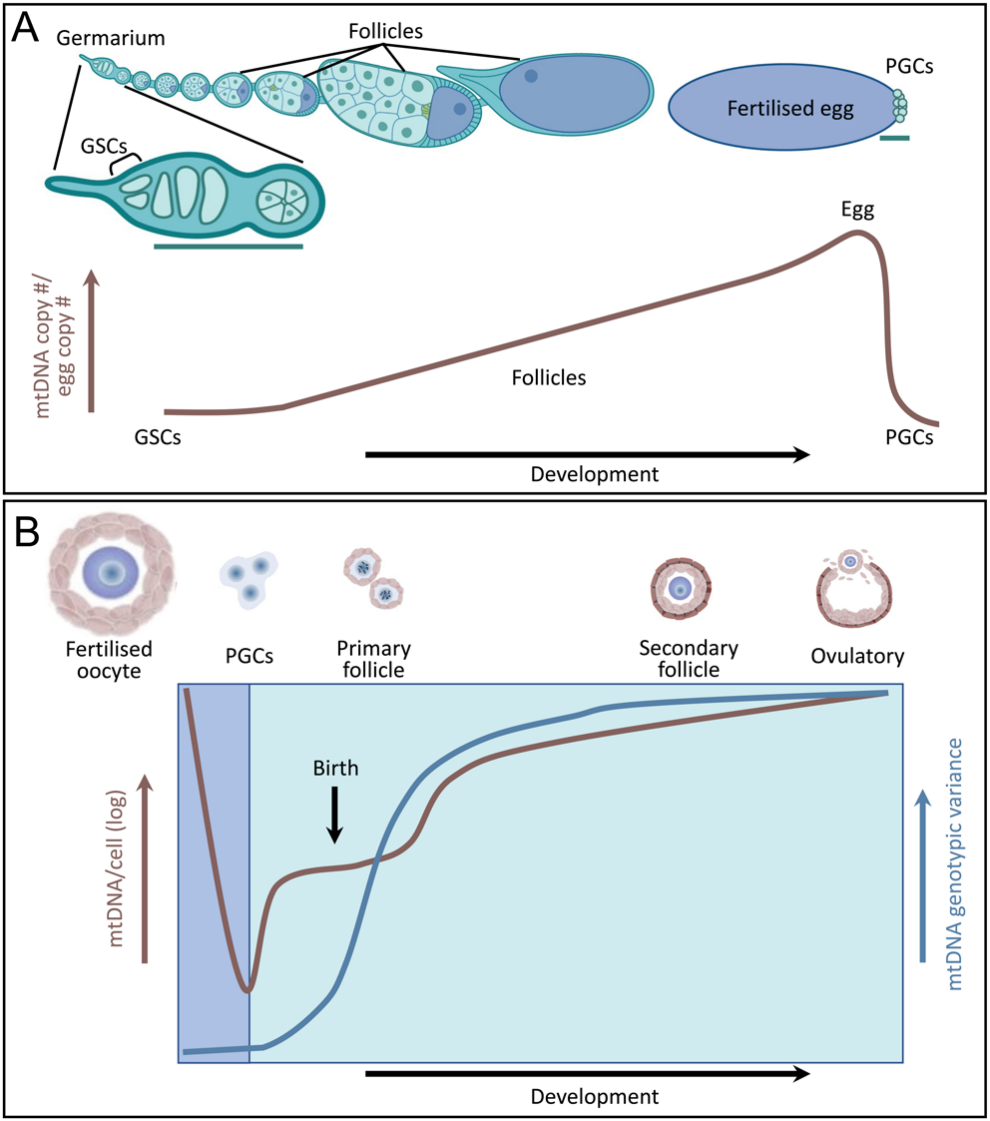
The mitochondrial bottleneck. (A) Mitochondrial DNA (mtDNA) copy number throughout Drosophila oogenesis. Cartoon of the ovariole at the top which contains developing follicles including the oocyte (blue) with a newly developing fertilized egg on the right. Germline stem cells (GSCs) are at the anterior of a specialized structure called the germarium. GSCs are present throughout the adult lifespan and continuously give rise to the germline. Primordial germ cells (PGCs) are the first cells formed at the posterior of the newly fertilized and deposited egg. The graph represents mtDNA copy number relative to the egg measured using quantitative mtDNA fluorescence *in situ* hybridization (FISH) and verified with qPCR for accessible stages (eggs, PGCs) (Hurd *et al.* 2016). mtDNA copy number greatly increases during follicle development then decreases when PGCs form. The green lines represent developmental time points that have been implicated in the genetic bottleneck due to decreased mtDNA copy number, mitochondrial dynamics, and mtDNA replication. (B) mtDNA copy number and genotypic variance throughout mammalian follicle development. Germ cell stages are indicated at the top. The profound drop in mtDNA copy number in PGCs followed by ~500-fold increase in copy number to mature oocyte enables clonal proliferation of mtDNA as well as passive selection of the best oocyte at the cellular level, the so-called 'ovarian bottleneck' (Wolf *et al.* 2017). Following fertilization, the oocyte divides and forms the inner cell mass of the blastocyst with little mtDNA replication (McConnell & Petrie 2004), where ~3 cells will develop into the embryo. In this 'postfertilization bottleneck' there may be active selection at the mtDNA level as well as passive compartmentalization. Evidence to date suggests that the major component of the variance in germline development arises prenatally during oogenesis (Li *et al.* 2016) and postnatally during folliculogenesis (Johnston *et al.* 2015).

Individual animals contain overwhelmingly WT mtDNA in their mitochondria, which is called homoplasmy. WT mtDNA mixed with mtDNA containing mutations, such as in a diseased state, or single nucleotide polymorphisms, such as in an experimental state, is called heteroplasmy. In addition to maternal inheritance, mitochondrial inheritance is driven by a developmental genetic bottleneck (Fig. 3) (Olivo *et al.* 1983). The general principle behind the genetic bottleneck is that heteroplasmic mothers produce offspring with ratios of WT to mutant mtDNA that are not the same as the mother. This rapid transgenerational segregation suggests that the number of segregating units is smaller than the 100,000 or so mtDNAs in the mammalian oocyte. Using allele-specific PCR, shifts in heteroplasmy have been used as a read out for pinpointing the developmental time points for the bottleneck (Johnston *et al.* 2015). In mouse and fly, this time point occurs very early in oogenesis and coincides in mouse with, but is not identical with, a reduction in mtDNA germ cell content (Cao *et al.* 2007, Wai *et al.* 2008) (Fig. 3). For mathematical modeling and cross-generational analysis, advances in mtDNA deep sequencing have been very useful to study the bottleneck (Arbeithuber *et al.* 2020). These types of analyses have been done for humans as well (Boucret *et al.* 2017).

In Drosophila, allelic-specific PCR and mtDNA Single Molecular Fluorescent *In Situ* Hybridization showed a steady increase in mtDNA copy number during oogenesis from GSCs to egg formation with a drastic decrease once the PGCs are formed at the beginning of embryogenesis (Fig. 3B) (Hurd *et al.* 2016). Examining mtDNA inheritance, studies identifying and analyzing mtDNA size variants indicated the larger size was preferentially transmitted as the heteroplasmic mothers aged (Solignac *et al.* 1984, 1987, Kann *et al.* 1998). A breakthrough occurred when researchers were able to create deleterious mtDNA mutations using mitochondria-targeted endonucleases (Srivastava & Moraes 2001, Xu *et al.* 2008). Using a temperature-sensitive mtDNA mutation and sequencing, Ma *et al.* found evidence for a genetic bottleneck early in oogenesis (Ma *et al.* 2014) (Fig. 3A). Using a different temperature-sensitive mtDNA mutation and imaging experiments to identify replicating mtDNA, Hill *et al.* found evidence supporting preferential replication of WT over mutant mtDNA could be responsible for the bottleneck (Hill *et al.* 2014). Recently, disruptions to fission and fusion have been shown to influence the bottleneck and inheritance, suggesting reducing organelle size and mtDNA content could be a mechanism supporting mtDNA inheritance and the genetic bottleneck (Lieber *et al.* 2019, Chen *et al.* 2020*b*). Interestingly, knocking down a*tg1* and *BNIP3* disrupted mtDNA selection, whereas *parkin* knockdown had no effect (Ma *et al.* 2014, Lieber *et al.* 2019). How maternal aging affects the bottleneck or mtDNA transmission in these models has not yet been investigated.

We and others demonstrated purifying selection against detrimental mtDNA mutants in mouse (Marchington *et al.* 1999), a process that may have evolved to maintain germline homoplasmy. However, purifying selection fails in human families with maternally inherited heteroplasmic mtDNA diseases and 'selfish' transmission of detrimental mtDNA is well established in flies (Klucnika & Ma 2019). The risks of transmitting disease to future children are poorly understood so reproductive decisions of potential parents are fraught with uncertainty. This is largely because of the mtDNA bottleneck. Indeed, high profile mtDNA replacement therapy has been developed in the UK as a reproductive option that aims to reduce this uncertainty (Poulton *et al.* 2019). While the time course of the mtDNA bottleneck is now emerging, the mechanisms are not clear, however, mitophagy is one of the processes that could contribute (Diot *et al.* 2016*a*). During transmission of mtDNA from mother to child, significant fluctuations in heteroplasmy are already apparent in mature oocytes (Marchington *et al.* 1997, Marchington *et al.* 1998) and this highlights the importance of understanding mitophagy in oocyte development.

Studies of artificially generated heteroplasmic mice suggest that mtDNA segregation occurs during oocyte development (Jenuth *et al.* 1996) and in preimplantation embryos between late morula and early gastrula (Latorre-Pellicer *et al.* 2019). Interactions with nuclear genes and metabolic factors can drive segregation, suggesting that mitochondrial fitness plays a role in the bottlenecking process (Lechuga-Vieco *et al.* 2020). Massive expansion in mtDNA content per cell occurs during development of PGCs to mature oocytes and provides an opportunity for mtDNA subpopulations to proliferate (Fig. 3B). Some authors have suggested that the segregation of pathogenic mtDNA mutations is non-random (Otten *et al.* 2018). Furthermore, the huge excess of oocytes in the ovary at birth compared with the numbers that are ovulated is a second opportunity for selection against poor mitochondrial subpopulations. However, the cellular mechanisms of purifying selection have barely been elucidated.

### MtDNA as a measure of mitochondrial quality in aging oocytes

A threshold level for mtDNA content has been associated with successful fertilization in mice (Wai *et al.* 2010) pigs (El Shourbagy *et al.* 2006) and cows (Lamas-Toranzo *et al.* 2018). However, whether a reduction in mtDNA content underlies the reduced success in oocytes from aged females remains unclear (Reynier *et al.* 2001, Chan *et al.* 2005, Murakoshi *et al.* 2013, Cree *et al.* 2015). Measurement of mtDNA copy number in single blastomeres from cleavage stage human embryos suggested that increased mtDNA copy number was associated with poor outcome and with oocytes from older mothers (Fragouli *et al.* 2013). However, this is controversial because data from other investigators does not support it (Murakoshi *et al.* 2013, Kim & Seli 2019). If so, the increased mtDNA copy number could be a compensation for a decline in mitochondrial function driven by aging.

Thousands of identical copies of mtDNA are present in most types of cells in normal individuals (homoplasmy), but in heteroplasmic mtDNA disease, normal and mutant mtDNA co-exist in the same cells. These findings soon suggested an mtDNA-based theory of aging in which an increase in heteroplasmic mtDNA point mutations and deletions (Cortopassi & Arnheim 1990) may underlie the decline in energy in aging individuals. The quality of mtDNA was directly implicated in a study in which multiple mtDNA mutations were generated in mice with deficient in the enzyme POLG (necessary for mtDNA synthesis and accurate replication) (Trifunovic *et al.* 2004). When maternally transmitted in mice, with a WT nuclear genome, such mtDNA mutations can induce mild aging phenotypes including impaired fertility (both litter size and number of litters) and shortened lifespan (Ross *et al.* 2013). Furthermore, accumulation of maternally transmitted mtDNA mutations down generations exacerbates this reduced fertility. Because oocytes contain a significant load of mutant mtDNA relative to controls in this model, mtDNA was explored in oocytes from aging women. However, the links between aging and oocyte mtDNA quality are by no means clear. Early investigators reported that they could detect mtDNA deletions in oocytes from older women using PCR (Chan *et al.* 2005), and findings were similar in cows (Hammond *et al.* 2016). However, these methods do not readily distinguish between deleted mtDNAs and other types of rearrangement such as mtDNA duplications. Once next-generation sequencing became available (Arbeithuber *et al.* 2020), germline heteroplasmy could be tested in more depth, but it is still not yet clear whether heteroplasmic point mutations increase in oocytes from aging women (Boucret *et al.* 2017) or cows (Hammond *et al.* 2016). Again, PCR was used to investigate arrested human embryos, and this suggested that mtDNA deletions were common. However, healthy embryos were not sampled so it remains unresolved as to whether mtDNA deletions are associated with poor outcome.

Other groups have investigated mtDNA heteroplasmy in normal human oocytes at different stages. Human PGCs can only be sampled from ovaries prenatally, so the data are scanty. At this stage, the mtDNA content may be as low as 200–2000 mtDNAs per cell and five mtDNAs per mitochondrion (Floros *et al.* 2018). The data are consistent with selection against non-synonymous mtDNA mutations prior to clonal expansion during oogenesis. In the later stages of oocyte development, cytoplasm adherent to the two polar bodies mitochondria can be sampled sequentially (De Fanti *et al.* 2017). While their data would be consistent with purifying selection between extrusion of the two polar bodies, the polar cytoplasm may be enriched in 'cellular garbage' and hence reflect the whole oocyte or zygote rather poorly.

In summary, while the mtDNA bottleneck appears to have evolved to improve mitochondrial quality in the offspring, it fails to eliminate mtDNA disease in humans and selfish mtDNA in flies. However, the physiological basis of the bottleneck is not clear and mitophagy could be important. There is little solid data demonstrating a decline in mitochondrial quality in oocytes from aging women and almost no published data on mitophagy during oocyte development.

## Unanswered questions and future directions

In conclusion, there are many gaps in our knowledge of how mitochondrial quality is maintained during oocyte development. While mitophagy is a cellular process that may well be involved, there is little if any hard data demonstrating its effects in oogenesis, oocyte development, and preimplantation development. Autophagy is clearly an essential process in early embryos in which transcription initiation must be accompanied by turnover of maternally inherited RNAs (Tsukamoto *et al.* 2008*a*) and autophagy-mediated apoptosis protects against aneuploidy (Singla *et al.* 2020). However, a role for mitophagy can only be inferred (Diot *et al.* 2016*a*). A group studying developmental programming were unable to detect mitophagy in oocytes, but their study was not exhaustive (Boudoures *et al.* 2017). Given that autophagy is required for maintaining healthy stem cells (Phadwal *et al.* 2013) and declines with aging, it is likely that the same is true of mitophagy. If mitophagy is required to maintain oocyte quality, it may also impact on our understanding of inherited mtDNA diseases and have implications for potential interventions such as mitochondrial replacement therapy. However, any connections between mitophagy and the decline in oocyte developmental potential in aging women remain conjectural.

Finally, the use of ARTs and cryopreservation for fertility preservation are being used ever more frequently, however, knowledge regarding the effect of these different modalities and processes on mitophagy is severely lacking. To address these gaps in our knowledge, in addition to studies using human reproductive tissues, which can be limited in supply, we should fully exploit valuable model organisms such as Drosophila and mouse. By adopting a multi-faceted strategy and using all available tools, we will move toward understanding the basic cell and molecular mechanisms governing mitophagy in aging germ cells and elucidate how ARTs influence mitophagy.

## Declaration of interest

The authors declare no conflict of interest. The funders had no role in the design of the study; in the collection, analyses, or interpretation of data; in the writing of the manuscript, or in the decision to publish the results. Suzannah Williams is a Lay Editor of Reproduction and Fertility. Suzannah Williams was not involved in the review or editorial process for this paper, on which she is listed as an author.

## Funding

Salary support for J P was from the NHS Highly Specialised services ‘Rare Mitochondrial Disorders of Adults and Children Diagnostic Service’. The views expressed are those of the authors and not necessarily those of the NHS.

## Author contribution statement

Conceptualization and writing were carried out by R T C, J P, and S A W. All authors have read and agreed to the published version of the manuscript.
